# Assessment of stress in patients with suicide attempts referred to the Emergency Poisoning Unit of Yazd in 2016

**DOI:** 10.4314/gmj.v56i2.10

**Published:** 2022-06

**Authors:** Rahele Jamali, Bonnie Bozorg, Hamid Owliaei, Reza Bidaki, Nasrolah Bashardoost, Soudabeh Keinia

**Affiliations:** 1 Ali-Ebn-Abitaleb Department of Medicine, Islamic Azad University, Yazd Branch, Yazd, Iran; 2 Shahid Beheshti University of Medical Sciences, Tehran, Iran; 3 Ali-Ebn-Abitaleb Department of Medicine, Islamic Azad University, Yazd Branch, Yazd, Iran; 4 Department of Psychiatry, Research Center of Addiction and Behavioral Sciences, Shahid Sadoughi University of Medical Sciences, Yazd, Iran; 5 Diabetes Research Center, Shahid Sadoughi University of Medical Sciences, Yazd, Iran; 6 Department of Epidemiology and statics, School of Medicine, Isfahan University of Medical Sciences, Isfahan, Iran; 7 Islamic Azad University, Yazd Branch, Yazd, Iran

**Keywords:** Suicide attempt, poisoning, depression, Iran

## Abstract

**Objective:**

Stress and life changes such as ageing, spouse's death, divorce, marriage, job loss, retirement, illness, interpersonal relationships and a history of suicide ideation or attempt may be considered risk factors for suicide attempts. This study aimed to investigate the relationship between stress and suicide.

**Design:**

Case-controlled and retrospective study.

**Participants:**

Patients referred to the Emergency Poisoning Unit of Yazd, were used as a case group and other admitted patients with various plans and etiologies were a control group, matched on age, gender, marital status and place of residence

**Settings:**

Patients were asked to fill out a questionnaire including age, gender, economic status, marital status, place of residence, a background of suicide, and history of family members' suicide as well as the Holmes-Rahe scale. SPSS 16, chi-square and t-test were used for analyzing data.

**Results:**

Comparing the Holmes-Rahe stress scale's scores of the case group (312.9±84.60) and control group (224.62±85.57) showed a significant statistical difference (P<0.0001). Regarding stress intensity, the score in the case group showed 6% mild stress, 40% moderate stress, 54% severe stress, and in the control group, 13%, 61% and 26%, respectively. Holmes-Rahe stress scale score of stress intensity showed a significant statistical difference between groups (P<0.0001).

**Conclusion:**

The results of this study suggest that stress was associated with increased suicide attempts.

**Funding:**

None declared

## Introduction

Life Stressors, transitions and changes such as ageing, spouse's death, divorce, job loss, retirement, illness, interpersonal relations conflicts, rejection and familial history of suicide can be considered risk factors for suicide attempts.^1^–^3^ Holmes and Rahe studied different life stressors and rated them; they showed that physical illness and psychiatric disorders increased following stressful event experiences.^4^ The Holmes-Rahe scale measures the mental and psychological burden of life changes and stressful life events. The events ranged based on their burden from the most to the least.^2^–^4^ Individuals exposed to many psychological problems and stressors may be prone to suicide ideation and attempt.^5^ Suicide is the act of intentionally causing one's death. The World Health Organization (WHO) statistics show that every year, 1 million people die from suicide, and suicide attempts are 20 times more than this rate.^6^–^7^ Fakhari showed that people with a suicide attempt have poorer relationships, problem-solving, roles, affective responses, and social interactions.

These groups use emotion-oriented styles or mechanisms and experience more stressful life events than the control group. Stressful events have a remarkable contribution in suicide attempts.^8^ Poursharifi et al. investigated the effects of stressors on deliberate suicide attempts with chemicals and found that the most common stressors are poverty and financial problems, attention seeking, unemployment, dispute with spouses and family conflicts.^9^ Mousavi, in his study, concluded that persons with suicide attempts had common personalities and traits such as introversion, distress, and psychosis; before attempting suicide, they had experienced more stressful events.^10^ In addition, they had elevated psychological distress and low resiliency, less use of mature problem-solving coping strategies, and lax religious attitudes.^11^We did not find a submitted study in this area in Iran.

Given the increased stress frequency of psychiatric disorders and suicide^12^, we investigated the relationship between stress and suicide in an Iranian patient sample. The stress diathesis model for suicide suggests the interaction of personality traits and environmental factors. Also, agents like poor social skills, cognitive errors, multiple psychic sufferings, pessimistic thoughts, hypothalamic-pituitary-adrenal axis stress-response system, and serotonin secretions defects are described.^13^

## Methods

### Study population

In this retrospective case-control study, the patients admitted to suicide attempts with various plans and etiologies to the Emergency Poisoning Unit of Yazd in 2015 comprised the case group with a suicide attempt. Another group of patients with no suicide were selected as a control group. All patients admitted because of a suicide attempt who met the inclusion criteria were included in this study. The participants in the control group were all patients admitted to the same ward who met the inclusion criteria and matched with the case group. The patients were informed about the study, and consent was provided.

### Study design

A case group with a suicide attempt and another group with no suicide attempts as a control group were selected. The case group was selected as available. All patients admitted for a suicide attempt and with inclusion criteria participated in this study. All patients were admitted to the same ward and had inclusion criteria for the control group, matched with the case group. The patients were informed about the study, and their consent was taken. The Cochran formula was used to calculate the sample size.

### Inclusion criteria

The age range included was 14–80, a history of suicide attempts by the patient or his/her family member and finally, a suicide attempt confirmed by emergency medicine specialists.

### Exclusion Criteria

All cases of suicide with no poisoning plan or poisoning without a suicide plan or no interest or consent for participation in the contribution of this study were excluded. The control group was matched with the case group based on age, gender, marital status, and place of residence.

Patients completed a checklist including age, gender, marital status, place of residence, background and cause of suicide, and suicide in family members besides the Holmes-Rahe stress scale. Forty-three questions must be answered yes or no, and the sum of scores was calculated. The least score was 11, and the most 481. For interpreting, scores more than 300 considered severe stress, more than or equal to 150 as moderate, and less than 150 as mild stress.^14^

### Instrument

Holmes-Rahe Life Stress Inventory^14^ assesses the extent of stress by 43 life events. The reliability of the test is based on Cronbach's alpha was 0.72, and its structural validity was estimated to be 0.82.^15^. Its reliability In the Iranian sample, 0.79 was reported through Cronbach's alpha method^16^ and its validity with the simultaneous validation method it has been reported with a stress index of 0.74.^17^ Finally, SPSS 16 was used and collected data analyzed via chi-square test and t-test. Homologizing age between groups was done by chi-square, and the result did not show any significant differences.

### Ethical approval

All subjects completed the informed consent form. The ethics committee and board of Shahid Sadoughi University of Medical Sciences of Yazd, Iran, authorized the study (Code: IR.SSU.REC.1394.006).

## Results

The mean age of the case group was 28.82±12.68, with a range of 14 to 80 years and the control group was 30.71±9.73, with a range of 14 to 80 years. Regarding gender, 42 participants in the case group were male and 58 females. In the control group, there were 59 females and 41 males

In the case group, there were 48 (48.5%) single and 51 (51.5%) married, chi-square was used to homologize groups, and the result showed no significant differences (p=0.669).

Regarding the place of residence, 93(93.9%) of partici-pants were urban, and the rest were rural. Applying chi-square to homologized groups showed no significant differences between case and control groups (p=0.132). Exploring the economic status of participants showed that 47(48.5%) were poor, 40(41.2%) were moderate, and 10 had good economic status. In the control group, 20 patients were poor, 63(63.6%) moderate, and the rest (10.3%) had a good economic status. The economic status parameter revealed a significant difference between the two groups (p<0.001).

Exploring the patient background of suicide, the case group showed that 11(11%) people had a suicide attempt in their history and 9 (9%) in their family members. In the control group, there were no suicide attempts in their background or among family members (Table 1). There was a significant difference between groups regarding the history of suicide (p=0.003, p=0.001, respectively).

**Table 1:** Differences in demographic variables between two groups

	Cases n (%)	Controls n (%)	P value
**sex**			
**Male**	58%	59%	0.866
**Female**	42%	41%	
**Marital status**			
**Married**	51.5%	54.5%	0.669
**Single**	48.5%	45.5%	
**Living Place**			
**State**	93.9%	96%	0.132
**Town**	6.1%	2%	
**Others**	0	2%	
**Economic status**			
**Poor**	48.5%	20.2%	**P<0.001**
**Moderate**	41.2%	63.6	
**Good**	10.3%	16.2%	
**Past history of suicide**			
**Yes**	11%	0%	**0.001**
**No**	89%	100%	
**Family history of** **suicide**			
**Yes**	9%	0%	**0.003**
**No**	91%	100%	

Exploring scores and stress intensity in groups showed stress scores based on Holmes-Rahe in case group 312.9 ±84.60 and in control group 224.62 ±85.57. The difference between the groups' stress scores based on Holmes-Rahe was statistically significant (p<0.0001). Also, exploring stress intensity in the case group revealed that 6% mild, 40% moderate and 54% severe stress among the group's participants. In comparison, in the control group, they were 13%, 61%, and 26%, respectively.

Stress intensity difference based on Holmes-Rahe was statically significant between groups (P<0.0001) (Table 2).

**Table 2:** Stress scores in groups based on Holmes-Rahe questionnaire

	Cases n (%)	Controls n (%)	P value
**Frequency** **based on stress** **intensity**			
**Mild**	6%	13%	**P<0.0001**
**Moderate**	40%	61%	
**Severe**	54%	26%	
**Stress score**			
**The least**	103	11	**P<0.0001**
**The most**	481	417	
**Mean ± SD**	84.60 ±312.9	85.57 ±224.62	

Investigation of the relationship among independent variables in this study with stress intensity showed that being female is significantly related to stress intensity. Still, such a relation wasn't seen between males and stress intensity (t=8.04, p<0.05). Considering age, in the range of 30–80, there was a significant correlation between age and stress intensity, but in the range of 14–24, there was no significant correlation respectively (r=0.7, p= 0.001), (r=0.21, p=0.137). It means older participants in age 30–80, experienced more stress events, but in ages ranged 14–24, this relationship is not substantial. The result showed a significant relationship between suicide attempts and stress intensity in married status and no significant relation in singles (r=0.83, p<0.0001, r= 0.11, p=0.267). There was no significant relationship between birth order and stress intensity (r=0.13, p=0.105). There were significant differences in stress intensity among different economic statuses (F=190.90 p=0.020). There was a significant relationship between stress intensity and suicide attempt in educational status at diploma level (r= 069, p=0.01), but no significant relationship between stress intensity and suicide attempt in educational status at the level of bachelor or higher and under diploma. Results showed a significant relationship between stress intensity and having a familial history of suicide (r= 0.79, p<0.0001). The cultural barriers, fear of disclosing identity data, and loss of response for some questions were subject to missing data.

## Discussion

This study aimed to investigate the relationship between suicide attempts and stress intensity of people who were referred to Yazd Emergency Poisoning Unit in 2016. This was a retrospective case-controlled study on 100 admitted patients in the Emergency Poisoning Unit in Yazd who participated as the case group, and 100 admitted patients with other reasons than suicide were selected as a control group.

As stress is the main factor in developing psychiatric problems, most families and patients never express it in their clinical interviews. It is important to investigate the relationship between stress and suicide, also the result of this study showed the stress score based on Holmes-Rahe in the case group and control group to be 312.9±84.60 and 224.62± 85.57, respectively and the mean differences between the two groups were statistically significant. In addition, the study showed a significant relationship between the female sex and stress intensity.

Saberi Zafarghandi and Mousavi showed that most of the people with suicide attempts were female, and our study also showed a significant relationship between stress intensity and being female. Therefore, we may conclude that stress may lead to more suicide attempts in women. 10–14, 18

In the range of 30–80 years old, there was a significant relationship with stress intensity, while there was no relation in the range of 14–29. A significant relation showed between being married and stress intensity, while there was no relation with being single. Regarding marital status in Saberi Zafarghandi and Mousavi study, being single was related to suicide attempts 10–14, 18 and also in our study, being married had a significant relationship with stress intensity. Therefore, it seems marriage is a transition in people's lives and can remarkably affect individuals' mental, behavioural, personality, and psychological aspects. Usually, married people are more promising and responsible in their jobs. Relations and effects on others are more important to them having children.

There was no significant relationship between the stress intensity and birth order (first, second, third, or more). A significant relation was seen between poor and moderate economic status and stress intensity, but there is no significant relationship between good economic status and stress intensity. Poverty may lead to less access to mental health care. The stress intensity and educational status at the diploma level were significant while no significant relation at the level of bachelor or higher and also under diploma. No history of suicide has a significant relationship with stress intensity.

The result of this study is congruent with Fakhari et.al in 2014. In Fakhari's study, individuals with suicide attempts have characteristics such as poor relationships, poor problem solving, affective involvement, affective responses and overall poor family performance. They were using emotion-oriented styles and experienced more stressful events in comparison with the control group. Fakhri's study showed the contribution of stressful events to suicide was the most (58%). They also indicated that economic, job, sexual and marriage stresses have a significant correlation with a suicide attempt.^18^ Moreover, Pour Sharifi et al. in 2012 showed that happiness, depression, stress and social support include 76% of suicide thinking.^9^

In the Shakeri et al. study in 2006, the most common factors of suicide attempts were poverty and financial problems, (64.75%), attention seeking (42.5%), unemployment (40%), dispute with spouse (32.5%) and conflict in the family(29.5%) respectively.^10^ In the male sex, common factors include poverty and financial problems and then unemployment and conflict in the family. However, in the female sex, they were getting attention, poverty and financial problems are more common and in youth, they were disputes with spouses, poverty and financial problems^10^ which were congruent with our study.

## Conclusion

The result of this study showed that stress and its severity are associated with suicide. It also revealed that there is a significant relation between stress intensity and being a female gender, marital status, being over 30 years old and poor economic status.
